# An examination of the interrelationships among NASA-TLX dimensions utilizing the DEMATEL method

**DOI:** 10.1371/journal.pone.0320638

**Published:** 2025-04-03

**Authors:** Şeniz Harputlu Aksu, Aylin Adem, Erman Çakıt, Metin Dağdeviren, Waldemar Karwowski

**Affiliations:** 1 The Scientific and Technological Research Council of Türkiye, Ankara, Türkiye; 2 Department of Industrial Engineering, Gazi University, Ankara, Türkiye; 3 Department of Industrial Engineering and Management Systems, University of Central Florida, Orlando, Florida, United States of America; Istanbul University: Istanbul Universitesi, TÜRKIYE

## Abstract

The NASA TLX is a survey-based method widely used to evaluate cognitive workload across six specific dimensions related to an employee’s tasks. While academic research recognizes that these dimensions may have unequal contributions to mental workload, they are often weighted using multi-criteria decision-making techniques. However, prior studies have not investigated possible relationships between the dimensions. The main objective of this paper is to explore the interconnections and dependencies among these dimensions. This study distinguishes itself by employing the Decision-Making Trial and Evaluation Laboratory (DEMATEL) technique to clarify these relationships and dependencies, and to examine how interactions between dimensions shift under different threshold conditions. The research includes three distinct impact diagrams tailored for individuals performing tasks with varying levels of cognitive workload. By considering the interdependencies among the NASA TLX dimensions, this study offers a significant advancement in the field, as these interrelationships could greatly influence the derived weights. This theoretical contribution has the potential to be groundbreaking in the realm of survey-based mental workload assessment techniques, such as the NASA TLX.

## 1. Introduction

The level of overload is essential for understanding the efficacy and well-being of employees. Considering this, researchers from various fields have made efforts to articulate constructs and devise instruments that allow for its thorough assessment. In recent years, the demands of business planning and coordination have predominantly become mental in nature. The concept of mental workload is particularly significant in the realm of ergonomic research and practice. As a result, the significance of understanding mental workload has grown. Mental workload is a “construct that reflects the relation between the environmental demands imposed on the human operator and the operator’s capabilities to meet those demands” [1]. The assessment of mental workload can be carried out using a variety of methods, which encompass (1) subjective assessments, (2) objective performance metrics, and (3) physiological or neurophysiological indicators [2]. Each category of measurement has its own unique set of benefits and drawbacks [3]. The user’s perceived mental workload can be evaluated through a questionnaire focused on subjective indicators.

Although there are multiple methods for assessing mental workload, the NASA Task Load Index (NASA-TLX) is among the most widely used in the field. The NASA-TLX scale, developed by Hart et al. [4], provides a thorough evaluation of workload. This scale encompasses six key dimensions: mental demand, physical demand, temporal demand, effort, personal performance, and frustration levels [5]. The implementation of the NASA-TLX requires participants to deliver two types of information concerning each dimension: weights and scores. The weights indicate the perceived significance of each dimension as a contributor to mental workload in the specific task, while the scores reflect the participants’ subjective assessment of the intensity of mental workload associated with each dimension [6]. Initially developed to address requirements in the aerospace sector, its application has broadened significantly over the years. Currently, the application of the NASA-TLX scale is not restricted solely to the aviation industry; it has found extensive application in multiple domains, including transportation, nuclear energy facilities, healthcare, and human-computer interaction, thereby becoming an indispensable tool for assessing workload in these areas [7,8]. Although the NASA-TLX is widely recognized, there is a significant lack of information and evaluations regarding the relationships and interdependencies among its various dimensions.

It is notable that even in recent years, both Raw NASA-TLX (where all dimensions are considered equally important) and the traditionally weighted NASA-TLX remain widely used in the literature [9–12]. Although efforts to introduce weighting through Multi-Criteria Decision-Making (MCDM) techniques have emerged, these studies generally do not extend beyond prioritization or ranking. This approach results in the neglect of potential causal relationships between dimensions. The present study was developed with the aim of addressing this gap, which is considered particularly significant in workload assessments across various sectors. The novelty of this paper lies in the fact that it applied the decision-making trial and evaluation laboratory (DEMATEL) technique to elucidate the relationships and dependencies between the dimensions of NASA-TLX, and it assessed how the interactions among these dimensions evolved with varying threshold values. Although this paper does not introduce new modifications to the DEMATEL method itself, its novelty lies in its integration with NASA-TLX, which has been enhanced through this combination. Specifically, NASA-TLX, a widely used tool for workload assessment, has been improved by incorporating DEMATEL to better identify and visualize causal relationships among workload factors. To our knowledge, no previous studies have applied this combined NASA-TLX and DEMATEL approach. Our research fills this gap by demonstrating how this integration offers deeper insights into the interdependencies of workload factors.

The organization of the following sections of this study is as follows: Section 2 presents a summary of the relevant subjective literature. Section 3 offers an in-depth introduction to the application of NASA-TLX and DEMATEL techniques. Section 4 discusses the implementation of the chosen MCDM techniques, along with a comparative analysis of the results. Finally, the concluding section outlines our findings and suggestions for future research.

## 2. Background

Despite the popularity and widespread adoption of NASA-TLX, the traditional method of weighting its subscales (mental demand, physical demand, temporal demand, performance, effort, frustration) remains contentious. Some have even suggested that weighting should be disregarded entirely. One such alternative, known as Raw-TLX, calculates the overall mental workload (MWL) index by taking the arithmetic mean of the dimension scores. While Raw-TLX offers a straightforward and efficient version of the original NASA-TLX, it is only appropriate when it is known in advance that each dimension is roughly equal in importance for a given task. However, this assumption does not always hold. Consequently, it is widely recognized that different subscales typically contribute to MWL in varying degrees, making it necessary to apply representative weights to reflect these differences. In this context, the traditional weighted NASA-TLX scale is commonly used in the literature. This weighting approach relies on the values obtained through pairwise comparisons of all sub-dimensions by the subject. However, this method has certain limitations regarding the assigned weights. Additionally, there are concerns that the timing of the subject’s comparisons may influence the scoring and prioritization process [6]. In this regard, there are some studies aiming to improve the practice of using NASA-TLX in a way that avoids the weighting issues inherent in the original version. In general, multi-criteria decision-making techniques with proven reliability and effectiveness are used in weighting the subscales of NASA-TLX. One of the most frequently used techniques is the Analytical Hierarchy Process (AHP) method. Eraslan et al. [13] evaluated the mental workload of academicians, administrative and technical staff working in a faculty with a scale they developed. They used the Fuzzy AHP method to give realistic results when making pairwise comparisons in subjective methods used to determine mental workload. Emeç and Akkaya [14] also used the Analytical Hierarchy Process (AHP) method and the NASA-TLX scale integratedly to determine the mental workload of 5 doctors working in a public hospital. In this study, unlike the classical method, they calculated the criterion weights with the AHP method. In another study, Gönen Ocaktan et al. [15] used the NASA-TLX method to determine the mental workload of employees in the sheet metal cutting process. They applied the fuzzy AHP method to determine the criterion weights. Virtanen et al. [6] suggested AHP and Swing methods as two alternative methods for criterion weighting in the NASA-TLX method. In this study, the outcomes of utilizing the original NASA-TLX, Swing, and AHP weighting methods within NASA-TLX and Raw-TLX were conceptually outlined and illustrated through data derived from 715 air combat simulator missions. The significance of employing weightings in NASA-TLX, particularly when Raw-TLX is deemed unsuitable, was examined, highlighting the advantages of the AHP and Swing weighting techniques in comparison to the traditional NASA-TLX weighting method from multiple viewpoints.

The NASA-TLX method was reconstructed by Çolak and Esen [16] using interval type-2 fuzzy sets. In the study conducted on 40 people working in the automotive industry, the interval type-2 fuzzy AHP method was used to determine the criterion weights, while a scale consisting of interval type-2 fuzzy numbers was used to score the jobs. Mouzé-Amady et al. [17] proposed a novel algorithm for calculating fuzzy estimates that offers a classification procedure responsive to different variables within work environments. This algorithm introduces a method for determining weights through qualitative fuzzy integrals and has been applied to the NASA-TLX subscales as an alternative to the traditional pairwise weighting technique (PWT). There are other studies that use fuzzy logic in deciding the importance order of subscales. Adar and Kılıç Delice [18] used the NASA-TLX method to determine the mental workload of research assistants working at the faculty of engineering and who were in the doctoral course, doctoral qualification and doctoral thesis stages, and used a decision model based on multi-member hesitant fuzzy linguistic expressions in calculating the weights of 6 factors of the method. Ekinci and Can [19] evaluated the ergonomic risk levels of 219 operators working in the fruit juice production process within the framework of the main criteria of perceived workload and working posture, with the help of the integrated multi-criteria decision making (MCDM) method. The CRiteria Importance Through Intercriteria Correlation (CRITIC) method was used to determine the importance weights of the criteria that are effective in the process of evaluating ergonomic risk levels, taking into account the perceived workload of the employees depending on the work they perform and the working postures they exhibit. Multi-Attributive Ideal Real Comparative Analysis (MIACRA) was applied to rank the employees according to their ergonomic risk levels. Kılıç Delice and Can [20] employed a methodology that combines the NASA-TLX and Stochastic Multi-Criteria Acceptability Analysis-2 (SMAA-2) methods to assess the mental workload of employees involved in the box letter manufacturing process. In this study, subprocesses across three distinct problem scenarios are ranked based on mental workload. The ranking results were the same in all three problem scenarios in question. As a result of this study, it was determined that the NASA-TLX&SMAA2 approach provides more distinct and clear results. Can [21] restructured the NASA-TLX method with intuitive fuzzy sets and used it to determine the mental workload of 153 industrial sales representatives. The NASA-TLX method is combined with intuitionistic fuzzy set (IFS) theory, creating an integrated approach called Intuitive Fuzzy TLX (IF-TLX). IFS theory serves as an effective tool for modeling uncertainty stemming from hesitation levels in human decision-making processes. The proposed method also took into account the effect of work experience in subjective workload evaluation.

Riono et al. [22] also used the NASA-TLX method together with fuzzy logic to measure the mental workload of ship personnel. Bandono and Riono [23] measured the mental workload of Indonesian warship workers using the fuzzy-based NASA-TLX method. As a result of the study, they determined that main machine operators had the highest mental workload, and electronic operators had the lowest mental workload. Malakoutikhah et al. [24] sought to enhance the NASA-TLX mental workload questionnaire by employing fuzzy linguistic variables in place of the traditional rating scale, and by utilizing the multi-criteria decision-making Fuzzy Best-Worst Method (FBWM) instead of the conventional pair-wise comparison approach. The findings indicated that the FBWM-NASA-TLX questionnaire was capable of providing more accurate assessments of workload. Wang et al. [25] (2021) employed the TOPSIS method as an alternative to the traditional weighted sum approach of the NASA-TLX for assessing mental workload. The model they proposed was tested using three distinct weighting strategies: fuzzy weighting, Hart weighting (which utilizes conventional pairwise comparisons), and uniform weighting. The efficacy of this method was assessed through an augmented reality user experiment. The relative closeness coefficient (RCC) derived from TOPSIS serves as a comprehensive metric for measuring and comparing workload levels across various virtual reality applications, demonstrating a lower coefficient of variation in subjective evaluations (CV=Standard Deviation/Mean) when contrasted with the classical weighted sum method. Therefore, it was found to be more reliable.

Table 1 presents the summary of the NASA TLX studies in the literature. In existing literature, studies often assume no interaction between the dimensions of NASA-TLX. Some studies determine the importance levels of these dimensions using traditional AHP or its fuzzy extensions, arguing that the dimensions do not contribute equally to the overall workload. However, such studies fail to consider the relationships between the dimensions. Calculating priority values or weights does not capture the interactions among them. In weight calculations, MCDM techniques generally operate under the assumption that the dimensions are independent and do not influence one another. However, the potential interactions between dimensions could affect the mental workload scores derived from NASA-TLX. The originality of this research arises from addressing this gap in the literature.

**Table 1:** The summary of the literature.

Author(s) (Year)	Sector	Number of Participants	MCDM Method	Fuzzy/Not Fuzzy
Mouzé-Amady et al. [17] (2013)	Aviation	53	Fuzzy logic	Fuzzy
Eraslan et al. [13] (2016)	Education	60	AHP	Fuzzy
Adar and Kılıç Delice [18] (2017)	Education	6	Multi-Criteria Hesitant Fuzzy Linguistic Term Set	Fuzzy
Ekinci and Can [19] (2018)	Manufacturing	219	CRiteria Importance Through Intercriteria Correlation-CRITIC and Multi Attributive Ideal-Real Comparative AnalysisMAIRCA	Not Fuzzy
Emeç and Akkaya [14] (2018)	Health	5	AHP	Not Fuzzy
Kılıç Delice and Can [20] (2018)	Manufacturing	12	Stochastic Multi-Criteria Acceptability Analysis-2 (SMAA-2)	Not Fuzzy
Can [21] (2018)	Manufacturing	153	Intuitionistic Fuzzy Set (IFS) Theory	Fuzzy
Riono et al. [22] (2018)	Defence	82	Fuzzy logic	Fuzzy
Bandono and Riono [23] (2019)	Defence	82	Fuzzy logic	Fuzzy
Malakoutikhah et al. [24] (2021)	Education	30	Fuzzy Best‑Worstmethod(FBWM)	Fuzzy
Wang et al. [25] (2021)	Education	15	TOPSIS (Fuzzy AHP weighting, Hart weighting (classical pairwise comparison) and uniform weighting)	Fuzzy
Gönen Ocaktan et al. [15] (2021)	Manufacturing	1	AHP	Fuzzy
Virtanen et al. [6] (2022)	Defence	16	AHP and Swing	Not Fuzzy
Çolak and Esen [16] (2023)	Automotive	40	Interval Type-2 Fuzzy Sets	Fuzzy

## 3. Methodology and application

### 3.1. NASA-TLX

In NASA-TLX method, developed by Hart and Staveland [26] to evaluate mental workload, six sub-criteria are used. These are mental demand (MD), physical demand (PD), temporal demand (TD), performance (PR), effort (EF) and frustration (FR). The PD dimension is assessed based on the level of physical activity required to complete the task. The dimension of MD shows how much space is given to mental activities such as thinking, decision making, research and perception during the task. In the dimension of TD, perceived time pressure and time stress while performing the task are evaluated. The PR dimension is evaluated how successful the task is to reach its goals. The EF dimension is about how much hard work is required to fulfill the mission. The FR dimension reflects levels of insecurity, dissatisfaction, disenchantment, and anger experienced during the task. The NASA-TLX comprises two parts. In the first part, each of the six dimensions is rated on a scale from 0 to 100, taking into account the work performed by the employee. The second part involves 15 pairwise comparisons to determine which dimension is the most significant contributor to workload. At the end of this comparison process, the relative weight of each dimension is calculated by counting the number of times it ranks first in a comparison, then dividing by 15 to normalize the result. The effect score of each dimension on overall workload (OWL) is then obtained by multiplying its relative weight by its rating. These effect scores are summed to produce a weighted total, which represents the OWL. This total, known as the TLX, ranges from 0 to 100 [18,21].

For instance, an evaluator (subject) assigned scores of 90, 5, 60, 30, 80, and 65 to the dimensions (MD, PD, TD, PR, EF, FR) on the NASA-TLX scale to assess perceived workload. When all sub-dimensions are assigned equal weight, the average score is calculated as **55**. Additionally, Table 2 presents the scores determined using the weights obtained through pairwise comparison of the sub-dimensions by the evaluator. Based on these weighted values, the total score was calculated as 61.67.

**Table 2:** Numerical example for NASA-TLX score calculation (with subject weighting).

	Scores given to sub-dimensions	In how many pairwise comparisons does it prevail?	Weights (by subject)	Weighted score
MD	90	1	0.07 (1/15)	6 (90x0.07)
PD	5	0	0.00 (0/15)	0 (5x0.00)
TD	60	3	0.20 (3/15)	12 (60x0.20)
PR	30	3	0.20 (3/15)	6 (30x0.20)
EF	80	3	0.20 (3/15)	16 (80x0.20)
FR	65	5	0.33 (5/15)	21.67 (65x0.33)
Total	55 (equally weighted)	15	1.00	61.67 (6 + 0 + 12 + 6 + 16 + 21.67)

Virtanen et al. [6] identified several challenges with the traditional weighting approach used in NASA-TLX. The first issue occurs when a person views two or more dimensions as equally important. Since the NASA-TLX weighting method requires a strict ranking in all pairwise comparisons, it does not allow individuals to express equal importance directly. The second, more significant challenge concerns inconsistency in these pairwise comparisons. If the true weights of the dimensions differ from the ideal ranking, individuals are forced to choose between accurately representing their perceived importance of each dimension or ensuring consistency in their comparisons. The third challenge involves the limited range of weights allowed by the method. Regardless of the pairwise comparison results, the possible range for each dimension’s weight is restricted to 0.00 to 0.33. This means it is impossible to assign a weight higher than 0.33, even if a dimension’s importance would justify a higher value.

As mentioned in the second section, many alternative weighting approaches have been used in the literature to overcome these challenges. It is noteworthy that in studies using NASA-TLX and MCDM techniques together, especially weighting with AHP is frequently used, however in-depth relationships and dependencies between sub-dimensions are ignored in this weighting process. To eliminate this gap in the literature, DEMATEL, one of the MCDM methods, was selected in this study to determine the importance levels of the sub-dimensions and cause-effect relationships and interactions between them.

### 3.2. DEMATEL (Decision-Making Trial and Evaluation Laboratory)

Each MCDM technique has unique characteristics and specific applications. Studies show that no single MCDA approach can address all decision problems. The choice of an MCDM method depends on the nature of the decision problem, the availability of relevant data, the complexity of the relationships, and the preferences of the decision makers. Nevertheless, DEMATEL stands out among MCDM techniques for its ability to capture detailed causal relationships and interdependencies between criteria [27]. Moreover, between factors and its calculation process is simpler and more straightforward [28]. The DEMATEL method was originally developed by Fontela and Gabus [29] in the 1970s, with the purpose of studying a complex and intertwined problem. It has been considered one of the best tools that can be used to unravel the cause-effect relationship between evaluation criteria [30].

**Fig 1:**
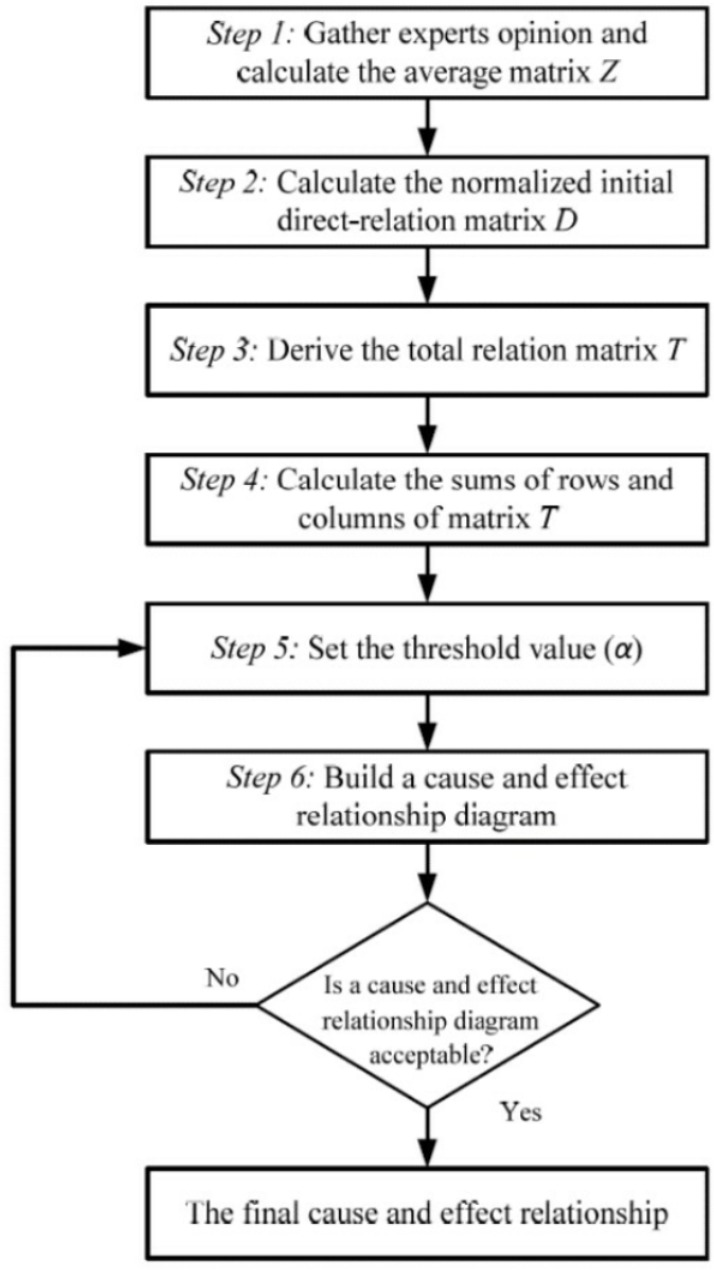
The process of the DEMATEL method.

As mentioned before, DEMATEL can transform relationships between factors into an understandable structural model of the system and separate the factors into a cause-and-effect group. Therefore, it is seen as a feasible and useful tool for analyzing the relationships between factors in a complex system and ranking those factors to make long-term strategic decisions. The steps of the classical DEMATEL method can be summarized as Fig 1 [30]. The full procedure of DEMATEL method is explained as follows [30,31]:

**Step 1** (generate the group direct-relation matrix *Z*). The direct-relation matrices are derived by performing pairwise comparisons among the criteria, where *z*_*ij*_ represents the extent to which criterion c_i_ influences criterion c_j_. Thus, the relationships among the criteria can be represented within a matrix. In this method, the impact of each criterion on the others is conveyed using linguistic expressions (no influence (0), low influence (1), medium influence (2), high influence (3), and very high influence (4). Initially, the individual direct-relation matrix *Z*_*k*_ =  [*z*_*ij*_^k^] _*n* ×  *n*_, as provided by the *k*th expert, is constructed. By synthesizing the opinions of the *l* experts, the group direct-influence matrix *Z* =  [*z*_*ij*_]_ × *n*_ can be derived through the following process:


$$z_{ij}=\frac{1}{l}\overset{l}{\underset{k=1}{\sum }}z_{ij}^{k}i,j=1,2,\ldots .,n.$$

**Step 2** (establish the normalized direct-relation matrix D). The normalized direct-relation matrix D =  [D_*ij*_]_ × *n*_ can be achieved by using:


$$\text{D}=\frac{Z}{s}$$

$$s=\text{max}(\text{max}\overset{n}{\underset{j=1}{\sum }}z_{ij},\text{max}\overset{n}{\underset{i=1}{\sum }}z_{ij})$$

**Step 3** (formulate the total-relation matrix *T*). The total-relation matrix T =  [t_*ij*_]_ × *n*_ is derived from the normalized direct-relation matrix D by aggregating both the direct effects and all indirect effects. This is expressed mathematically as *T* =  D + D^2^ + D^3^ +  ⋅  ⋅⋅ +  D^ℎ^ =  D(*I* −  D)^−1^, as ℎ →∞, where *I* represents the identity matrix.

**Step 4** involves the creation of the influential relation map (IRM). In this phase, the vectors *R* ([r_*i*_]_ × 1_) and *C* ([c_j_]_1 × n_) are established, which correspond to the summation of the rows and the summation of the columns from the total-relation matrix *T*, respectively, as defined by the subsequent formulas:


$$R=\overset{n}{\underset{j=1}{\sum }}\left(\text{tij}\right)$$

$$C=\overset{n}{\underset{i=1}{\sum }}\left(\text{tij}\right)$$

The sum (*R* +  *C*), referred to as “Prominence,” represents the degree to which a factor plays a central role in the system. Similarly, the vertical axis vector (*R* −  *C*), known as “Relation,” indicates the net effect a factor has on the system. If (*rj* −  *cj*) is positive, factor *j* exerts a net influence on other factors and should be categorized in the cause group; if (*rj* −  *cj*) is negative, factor *j* is primarily influenced by other factors and belongs in the effect group.

**Step 5 (determine criteria weights). The weight of each criterion is calculated as follows:**


$$\text{wj}=\frac{\sqrt{{\left(\text{rj}+\text{cj}\right)}^{2}+{\left(\text{rj }-\text{cj}\right)}^{2}}}{\sum_{j=1}^{n}\sqrt{{\left(\text{rj}+\text{cj}\right)}^{2}+{\left(\text{rj }-\text{cj}\right)}^{2}}}$$

**Step 6** (set the threshold value). In certain situations, the IRM can become too complex to effectively display valuable information for decision-making if all relationships are included. To address this, many studies set a threshold value to filter out minor effects. In other words, only elements in matrix *T* with an influence level above the threshold are selected and converted into an IRM.

An excessively high threshold value reduces the weight of expert opinions and oversimplifies the issue, while a threshold that is too low results in divergent perspectives and a lack of focus. When the threshold is set too low, numerous factors are included, making the IRM overly complex and hard to interpret. On the other hand, if the threshold is set too high, some critical factors may be excluded. Therefore, if the threshold value does not effectively differentiate expert opinions, it cannot accurately capture the key factors of a complex problem [32]. Therefore, different threshold values can be applied until acceptability of effect relationship diagram is achieved. The literature indicates that experts commonly set the threshold value through discussions [33–36]. However, some researchers apply different strategies to determine this threshold, such as the brainstorming technique [37], maximum mean entropy (MMDE) [38,39], the average of all elements in matrix *T* [40], or the maximum value of the diagonal elements in matrix *T* [41].

We applied the DEMATEL method to determine the relationships among the dimensions, as it is particularly well-suited for evaluating complex interdependencies and causal relationships between multiple criteria. DEMATEL’s ability to identify both direct and indirect relationships between variables makes it an ideal choice for our study, where understanding the interactions between different factors is essential. This method provides a more comprehensive understanding of the system’s structure. While various techniques exist in the literature for analyzing relationships among system elements, the following considerations influenced our decision to use DEMATEL. For instance, the Analytical Network Process (ANP) requires prior knowledge of relationships between dimensions for effective application. Therefore, we first employed the DEMATEL technique to identify these relationships.

Structural Equation Modeling (SEM) is another approach for modeling relationships and testing hypotheses. However, it typically requires extensive data and large sample sizes [42], which can be a challenge in certain research contexts, including ours. While SEM is well-suited for continuous data and can handle categorical data with appropriate techniques, DEMATEL offers greater flexibility, effectively working with both qualitative and quantitative data. This makes it a more versatile choice for complex decision-making scenarios [43].

Similarly, Interpretive Structural Modeling (ISM) is a tool used to identify cause-and-effect relationships among decision elements. However, ISM primarily relies on binary inputs (0,1) and is generally used to extract the hierarchical structure of factors [44]. In contrast, our study aims to determine both the direction and intensity of interactions between factors. Considering these factors, we selected the DEMATEL technique, as it clearly identifies cause-and-effect relationships among variables. By utilizing DEMATEL, we were able to develop a more robust and dynamic model of interactions.

## 4. Application and results

In the application section, the steps of the DEMATEL method explained above were applied for NASA-TLX sub-dimensions.

**Step 1.** Firstly, initial direct-relation matrix Z was established as Table 3. All principal diagonal elements are equal to zero. The direct relationship matrix included in this study was obtained through a joint evaluation by ergonomists and occupational safety experts with experience in the private sector, as well as academics who have conducted studies on mental workload. Consent forms were not obtained because the data were analyzed anonymously. The direct relationship matrix in this study was developed through a collaborative assessment conducted by a panel of experts chosen for their relevant experience and expertise. This panel included ergonomists and occupational safety specialists with extensive private sector experience, particularly in evaluating both mental and physical workloads. Additionally, academics specializing in mental workload research, with contributions to the field through empirical and theoretical studies, were also involved. Experts were selected based on three key criteria: professional experience (at least 10 years in ergonomics, occupational safety, or workload analysis), academic contributions (researchers with published studies on mental workload or related topics in reputable journals), and diversity of perspectives (a balanced mix of industry professionals and academics to incorporate both practical and theoretical insights). The expert panel comprised three decision-makers: one ergonomist and occupational safety expert, along with two academics. Their insights were gathered through structured workshops where they collaboratively assessed and discussed the interactions between various dimensions. A consensus-based approach was used to ensure agreement on the relationships within the direct relationship matrix.

**Table 3:** The group direct-relation matrix *Z.*

	MD	PD	TD	PR	EF	FR
MD	0	1.2	0	2.1	2.7	2.3
PD	1.4	0	1.6	3.4	3.2	3.8
TD	3.7	3.6	0	3.2	3.3	3.6
PR	1.3	2.2	2.8	0	3.1	3.7
EF	2.4	2.7	1	3.3	0	3.6
FR	1.1	1.7	0.4	1	2.3	0

**Step 2.** The value of s was calculated. The s value was found to be approximately 0.0575 with the calculation of 1/17.4. By using Z matrix, the normalized direct-relation matrix D was generated as below (see Table 4).

**Table 4:** The normalized direct-relation matrix D.

	MD	PD	TD	PR	EF	FR
MD	0	0.068966	0	0.12069	0.155172	0.132184
PD	0.08046	0	0.091954	0.195402	0.183908	0.218391
TD	0.212644	0.206897	0	0.183908	0.189655	0.206897
PR	0.074713	0.126437	0.16092	0	0.178161	0.212644
EF	0.137931	0.155172	0.057471	0.189655	0	0.206897
FR	0.063218	0.097701	0.022989	0.057471	0.132184	0

**Step 3.** The total-relation matrix *T* was established by using the formulation of D (I −  D)^−1^ (see Table 5).

**Table 5:** The total-relation matrix *T.*

	MD	PD	TD	PR	EF	FR
MD	0.123401	0.21465	0.090534	0.275337	0.324948	0.339884
PD	0.279371	0.24653	0.224393	0.438119	0.465252	0.545007
TD	0.441705	0.483774	0.171825	0.504247	0.552607	0.628042
PR	0.281042	0.362168	0.277261	0.274176	0.461183	0.53997
EF	0.311286	0.364094	0.18694	0.418035	0.293378	0.515827
FR	0.165767	0.21542	0.09523	0.200289	0.276171	0.188389

**Step 4.** After the calculation of T matrix, R, C, (R + C) and (R-C) matrices were calculated. Table 6 shows values of these matrices.

**Table 6. R:** C, (R + C) and (R-C) matrices.

	R	C	R + C	R-C
MD	1.368754	1.602572	2.971326	-0.23382
PD	2.198672	1.886635	4.085308	0.312037
TD	2.7822	1.046182	3.828382	1.736017
PR	2.195801	2.110204	4.306005	0.085597
EF	2.089559	2.373539	4.463098	-0.28398
FR	1.141267	2.75712	3.898386	-1.61585

The PD, TD, and PR sub-dimensions, where (R-C) has positive values, exhibit a net influence on the other factors and can be classified as the cause group. Conversely, MD, EF, and FR are influenced by the other factors and should be classified as the effect group.

**Step 5.** After calculating the (R + C) and (R-C) matrices, the criteria weights were also determined. The results indicate that the sub-dimension with the highest weight is EF, while the sub-dimension with the lowest weight is MD (Table 7).

**Table 7:** Criteria weights.

	w
MD	0.122754
PD	0.168746
TD	0.173128
PR	0.177381
EF	0.184187
FR	0.173803

**Step 6.** To observe the variation based on different threshold values, three distinct thresholds were evaluated. First, a threshold of 0.3271—representing the average of the values in the T matrix—was selected. Values exceeding this threshold are highlighted in color in the T matrix shown in Table 8.

**Table 8:** T matrix colored based on the first threshold value (threshold: 0.3271) (*).

	MD	PD	TD	PR	EF	FR
MD	0.123401	0.21465	0.090534	0.275337	0.324948	0.339884
PD	0.279371	0.24653	0.224393	0.438119	0.465252	0.545007
TD	0.441705	0.483774	0.171825	0.504247	0.552607	0.628042
PR	0.281042	0.362168	0.277261	0.274176	0.461183	0.53997
EF	0.311286	0.364094	0.18694	0.418035	0.293378	0.515827
FR	0.165767	0.21542	0.09523	0.200289	0.276171	0.188389

(*) Orange-colored criterion names indicate dominant criteria, and green-colored numbers indicate values above the threshold.

**Fig 2:**
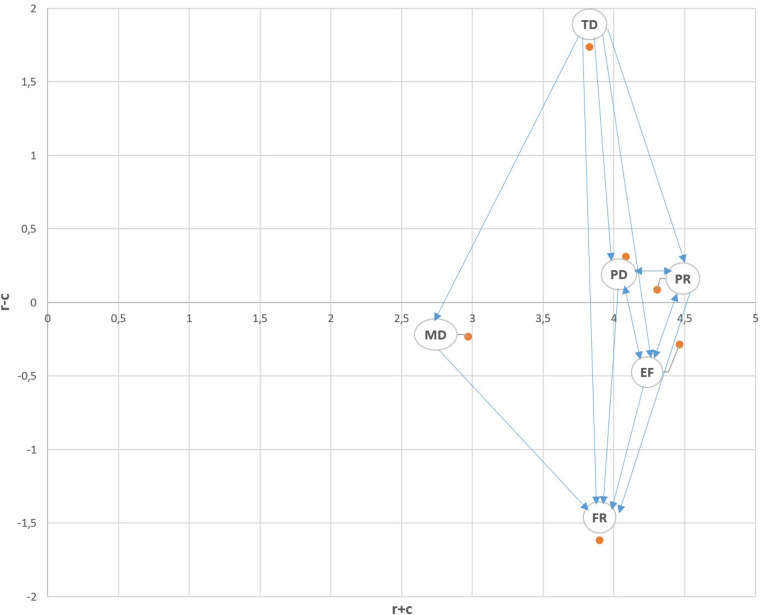
IRM based on the first threshold value (threshold: 0.3271).

An influential relation map (IRM) was drawn according to these colored values in the T matrix of Table 8 (see Fig 2). The factors that remain in the positive area (PD, TD, and PR) are dominant factors. It is noteworthy that TD affects all sub-dimensions except itself, while FR is affected by all sub-dimensions except itself. Although TD affects all sub-dimensions except itself, it is not affected by any sub-dimensions. Similarly, although FR is affected by all sub-dimensions except itself, it cannot affect any sub-dimensions including itself.

**Fig 3:**
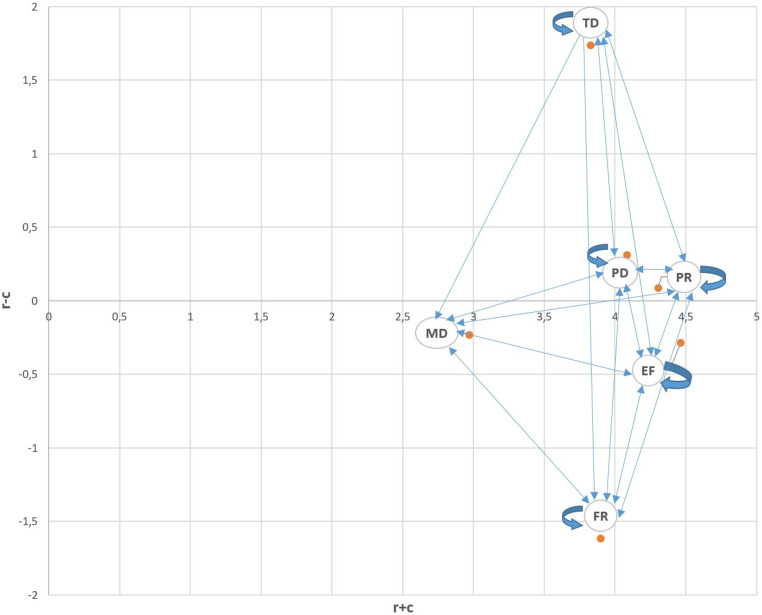
IRM based on the second threshold value (threshold: 0.15).

For the second case where the threshold value is taken as 0.15 and the third case where the threshold value is taken as 0.45, the T matrices are colored as in Tables 9 and 10. When the threshold value is lowered, it is seen that almost all values of the T matrix remain above the threshold. Therefore, many factors have been included in the graph diagram and IRM has become much more complex than the previous one (see Fig 3). Based on these relationships, it can be interpreted that the jobs in this group consist of complex work areas such as surgery, air traffic control, and software development, which require a much higher mental workload. According to this IRM, it is seen that TD is still the least affected sub-dimension.

**Table 10:** T matrix colored based on the third threshold value (threshold: 0.45) (*).

	MD	PD	TD	PR	EF	FR
MD	0.123401	0.21465	0.090534	0.275337	0.324948	0.339884
PD	0.279371	0.24653	0.224393	0.438119	0.465252	0.545007
TD	0.441705	0.483774	0.171825	0.504247	0.552607	0.628042
PR	0.281042	0.362168	0.277261	0.274176	0.461183	0.53997
EF	0.311286	0.364094	0.18694	0.418035	0.293378	0.515827
FR	0.165767	0.21542	0.09523	0.200289	0.276171	0.188389

(*) Orange-colored criterion names indicate dominant criteria, and green-colored numbers indicate values above the threshold.

**Table 9:** T matrix colored based on the second threshold value (threshold: 0.15) (*).

	MD	PD	TD	PR	EF	FR
MD	0.123401	0.21465	0.090534	0.275337	0.324948	0.339884
PD	0.279371	0.24653	0.224393	0.438119	0.465252	0.545007
TD	0.441705	0.483774	0.171825	0.504247	0.552607	0.628042
PR	0.281042	0.362168	0.277261	0.274176	0.461183	0.53997
EF	0.311286	0.364094	0.18694	0.418035	0.293378	0.515827
FR	0.165767	0.21542	0.09523	0.200289	0.276171	0.188389

(*) Orange-colored criterion names indicate dominant criteria, and green-colored numbers indicate values above the threshold.

**Fig 4:**
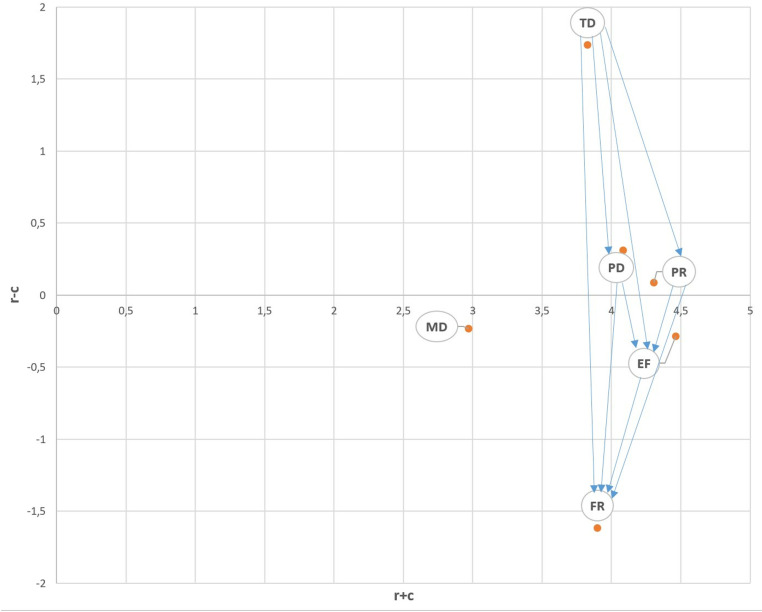
IRM based on the third threshold value (threshold: 0.45).

The values in the T matrix are recolored according to the third case, where the threshold value is 0.45 (see Table 10). The number of interactions decreased for this case. IRM has also become simpler (see Fig 4). Based on these relationships, it can be interpreted that the jobs in this group consist of simple office duties or call center tasks, which require a low mental workload. This graph is meaningful in revealing the most important interactions.

In this case, the MD sub-dimension is not affected by any dimension and does not affect any of them. The factor that affects more sub-dimensions than the others is TD, and the factor that is affected by the largest number of sub-dimensions is FR. TD affects PD, PR, EF and FR. FR is affected by PD, TD, PR and EF. Physical demand and performance affect effort and frustration. Effort is also effective on frustration. Time pressure (temporal demand) affects both physical demand, performance, effort and frustration. According to these results, it is possible to say that the most dominant sub-dimension in the NASA-TLX scale is temporal demand (TD). TD is the leading member of the cause group and FR is the leading member of the effect group.

When the total score for the numerical example presented in the NASA-TLX section was recalculated using DEMATEL weights, a value of 53.63 was obtained, as shown in Table 11. The result closely aligns with the score calculated using the Raw NASA-TLX method. However, a slight decrease in the total score is observed due to the low weighting of the MD criterion by DEMATEL, despite it receiving the highest score from the subject. Similarly, the contributions of sub-dimensions such as EF, PR, FR, and TD—identified as significant by DEMATEL—can also be seen in the total score.

**Table 11:** Numerical example for NASA-TLX score calculation (with DEMATEL weighting).

	Scores given to sub-dimensions	DEMATEL Weights	Weighted score (weighted with DEMATEL)
MD	90	0.122754	11.05 (90x0.122754)
PD	5	0.168746	0.84 (5x0.168746)
TD	60	0.173128	10.39 (60x0.173128)
PR	30	0.177381	5.32 (30x0.177381)
EF	80	0.184187	14.73 (80x0.184187)
FR	65	0.173803	11.30 (65x0.173803)
Total	**55** (equally weighted)	1.00	**53.63** (11.05 + 0.84 + 10.39 + 5.32 + 14.73 + 11.30)

The proposed model in this study was not tested within a specific organization. Instead, the direct relationship matrix was developed through a joint evaluation by ergonomists and occupational safety experts, ensuring a well-founded and comprehensive theoretical framework. This approach enables us to make a theoretical contribution to the literature by offering a methodological foundation that can be adapted and applied across various organizational settings. However, illustrative examples have been included to demonstrate the model’s applicability. Our primary objective in this study was to establish a strong theoretical foundation and showcase the potential of our framework for analyzing complex interactions, particularly in the context of workload and decision-making processes. To achieve this, the traditional NASA-TLX technique was extended using the DEMATEL method.

Although the DEMATEL technique theoretically allows for weight calculation, it is generally not the preferred method for weight determination in the literature. Instead, it is typically used in conjunction with other MCDM techniques, such as ANP [45–48]. As previously mentioned, the primary function of DEMATEL is to identify the interactions between decision-making elements and determine which elements exert influence and which are influenced.

In this study, the relationships between NASA-TLX dimensions are analyzed using various threshold values. The objective is to emphasize that, before applying NASA-TLX in a sequential manner, the network of relationships among the dimensions should be considered, ensuring that evaluations account for these interdependencies.

## 5. Discussion and Conclusion

Although alternative methods exist, NASA TLX remains a widely used survey-based technique in both research and practical applications for assessing mental workload. This approach includes six dimensions to evaluate the tasks performed by employees. Academic studies recognize that each dimension may contribute differently to mental workload and have applied weights to these dimensions, typically through multi-criteria decision-making techniques. Numerous studies in the literature have utilized AHP and other MCDM techniques, including their fuzzy extensions, to determine the weights of NASA-TLX dimensions. However, this paper seeks to move beyond correlation analysis by applying DEMATEL to uncover causal relationships and interactions among these dimensions. Unlike conventional methods that primarily assess the strength and direction of linear relationships, DEMATEL enables an exploration of the system’s underlying structure by identifying both direct and indirect influences. Specifically, the primary objective of this study is not to establish a hierarchical ranking but to illuminate the intricate interplay and feedback loops among the dimensions. This approach aligns with the core philosophy of DEMATEL, which is recognized as a powerful method for identifying cause-and-effect relationships within complex systems. By analyzing interdependent factors and visualizing their structural connections, DEMATEL helps pinpoint key elements in a system [42]. For example, using DEMATEL, we can distinguish between causal factors and those that are primarily influenced, offering insights that traditional correlation methods cannot provide.

A close examination of the NASA TLX dimensions (mental demand, physical demand, temporal demand, performance, effort, and frustration) reveals that these criteria influence each other. However, simply analyzing the criteria is insufficient to determine the direction and extent of these interactions. An analytical approach is required to understand both the nature and direction of these relationships. With this in mind, the study employed the DEMATEL technique to analyze the interactions between the NASA TLX dimensions. It was specifically considered that adjusting the threshold values—a core aspect of the DEMATEL method—would impact these relationships. The study presents three different impact diagrams for individuals engaged in tasks with varying mental workload levels. Clearly, it would not be realistic to use a single influence diagram for employees in high-attention roles like air traffic control, surgery, or software coding, and those in roles like call centers, accounting, or secretarial work. Although individuals in these professions may respond differently to surveys, the results are ultimately shaped by the interrelationships among the criteria. Differentiating between these roles in this manner enhances the realism of the findings. This study accounted for this need by proposing distinct patterns for different types of work requiring varying levels of mental workload, using adjusted threshold values.

Since this study focuses exclusively on the impact of DEMATEL-derived criterion weights, the numerical example compares equal weighting with DEMATEL weighting. The validity and utility of Raw NASA-TLX for workload assessment are evident. A review of the numerical example included in the study confirms that the DEMATEL-integrated NASA-TLX calculation yields consistent results. However, as emphasized in this research, DEMATEL offers additional insights into sector-specific differences and relationships between criteria. While obtaining similar results reinforces consistency, the true advantage of this approach lies in its ability to interpret causality between dimensions under varying threshold values.

As the threshold level decreases, the values of the dimensions that influence and are influenced by each other will rise, adding to the overall complexity. This is especially applicable to tasks that demand a high cognitive load, making the findings for these roles more realistic. Conversely, raising the threshold level will simplify the graphs of NASA TLX dimensions, reducing the degree of interaction. This approach can serve as an initial assessment for jobs with low cognitive load, such as very simple routine office tasks.

For instance, if a threshold value of 0.45 is applied to a scenario where perceived workload is measured during a routine office task, the MD factor—if it neither influences nor is influenced by other factors in this scenario—could be disregarded. Once the MD factor is removed and the DEMATEL weights are redistributed among the remaining dimensions, the total score in the numerical example further decreases to 48.54. This refined approach may lead to a more realistic assessment of workload in routine office settings, where the level of strain is not excessively high.

While this study enhances the NASA TLX scale in several ways, it does have certain limitations. The primary limitation relates to its theoretical contribution to the literature. Future research could compare the proposed scale with the traditional NASA TLX scale in specific real-life applications. The main objective is to highlight the presence of interactions between NASA-TLX dimensions and to theoretically demonstrate that effect graphs, shaped by adjusting threshold values, can vary. Our study provides a new perspective on the standard NASA-TLX technique and, unlike previous research, underscores the significance of relationships between dimensions. The theoretical contribution of this study has the potential to transform mental workload measurement techniques.

## Supporting information

S1 Data Raw data. (XLXS)
